# Effectiveness and Cost of Insecticide-Treated Bed Nets and Indoor Residual Spraying for the Control of Cutaneous Leishmaniasis: A Cluster-Randomized Control Trial in Morocco

**DOI:** 10.4269/ajtmh.14-0510

**Published:** 2016-03-02

**Authors:** Chafika Faraj, Joshua Yukich, El Bachir Adlaoui, Rachid Wahabi, Abraham Peter Mnzava, Mustapha Kaddaf, Abderrahmane Laamrani El Idrissi, Btissam Ameur, Immo Kleinschmidt

**Affiliations:** Laboratoire d'Entomologie Médicale, Institut National d'Hygiène, Rabat, Morocco; Center for Applied Malaria Research and Evaluation, Tulane University School of Public Health and Tropical Medicine, New Orleans, Louisiana; Directorate of Epidemiology and Disease Control, Ministry of Health, Rabat, Morocco; London School of Hygiene and Tropical Medicine, London, United Kingdom

## Abstract

Cutaneous leishmaniasis (CL) remains an important public health problem in Morocco. A cluster-randomized trial was conducted with the following three study arms: 1) long-lasting insecticide-treated nets (LLINs) plus standard of care environmental management (SoC-EM), 2) indoor residual spraying (IRS) with α-cypermethrin plus SoC-EM, and 3) SoC-EM alone. Incidence of new CL cases by passive and active case detection, sandfly abundance, and cost and cost-effectiveness was compared between study arms over 5 years. Incidence of CL and sandfly abundance were significantly lower in the IRS arm compared with SoC-EM (CL incidence rate ratio = 0.32, 95% confidence interval [CI] = 0.15–0.69, *P* = 0.005 and sandfly abundance ratio = 0.39, 95% CI = 0.18–0.85, *P* = 0.022). Reductions in the LLIN arm of the study were not significant, possibly due to poor compliance. IRS was effective and more cost-effective for the prevention of CL in Morocco.

## Introduction

Human infection by *Leishmania* spp. is an important public health problem in Morocco.^1^–^3^ Transmission of *Leishmania* parasites and the resulting disease is endemic throughout many areas of the country with three distinct parasites and disease patterns, typically divided into distinct bioclimactic zones.^3^,^4^ Anthroponotic cutaneous leishmaniasis (ACL) caused by *Leishmania tropica* and zoonotic cutaneous leishmaniasis (ZCL) caused by *L. major* are the most prevalent manifestations of the disease, however, the presence of zoonotic visceral leishmaniasis (ZVL) caused by *L. infantum* has been recognized since the 1920s.^5^ *L. infantum* has also been demonstrated in cutaneous lesions in Morocco.^6^–^8^ The parasites are transmitted by Phlebotomine sandflies, namely *Phlebotomus sergenti* and *Ph. papatasi* for ACL and ZCL, respectively.^2^ The main reservoir host for *L. major* is considered to be the rodent *Meriones grandi*, the Moroccan jird.^2^

Major epidemics of ACL and ZCL have occurred in Morocco recently, with the number of cases of cutaneous leishmaniasis (CL) caused by *L. major* and *L. tropica* in 2010 reaching over 8,000 nationwide and cases emergent in new areas previously believed absent of either disease.^4^ For this reason, the national vector control program has sought to expand and improve preventative control of the disease through expansion and intensification of vector control efforts. The standard of care for prevention of CL transmission in Morocco has been environmental management (EM) including promotion of improved solid waste disposal practices, and the promotion of local plastering or sealing of cracks and crevices in walls and animal shelters. However, there is evidence showing that, in the presence of endophagic or endophillic leishmaniasis vectors, insecticide-treated bed nets (ITNs) and indoor residual spraying (IRS) may be effective means of prevention of transmission of *Leishmania* spp.^9^ Trials and observational studies of both strategies have shown mixed results, possibly due to a number of factors including the mesh size of nets used, the susceptibility of the vector to insecticide, biting behavior of the vectors, and the quality of application of the intervention.^9^

A cluster-randomized control trial in Bangladesh and Nepal found statistically significant reductions in vector density with three interventions IRS, ITNs, and EM (filling cracks and crevices) for the vector *Ph. argentipes*, the effect size in the trial for IRS was the strongest (> 70% reduction in sandfly density), with ITNs and EM showing similar, but smaller reductions (∼40%).^10^ A second cluster-randomized control trial in India and Nepal demonstrated an approximately 25% reduction in the density of *Ph. argentipes* with ITN use.^11^ An individual (household) block-randomized trial in an urban area (Kabul, Afghanistan), where the predominant vector is *Ph. sergenti*, found a strong protective effect against ACL with the use of ITNs, chadors (wraps/top sheets), and IRS. ITNs and chadors showed the largest effects.^12^ Three small trials in Syria, where *Ph. sergenti* is the main vector of ACL, showed significant impacts of ITN use versus either untreated nets or no intervention, including on confirmed CL incidence.^13^,^14^ A study conducted in Khartoum, Sudan demonstrated decreased survival of *Ph. papatasi* collected from rooms with ITNs or insecticide-treated curtains as compared with those collected from untreated control rooms and three small studies in Iran, including one cluster-randomized trial, in areas where *Ph. sergenti* is the dominant vector, demonstrated reductions in CL incidence following ITN distribution either compared with no intervention or to untreated nets.^15^–^18^ Finally, a study in Turkey also demonstrated a decrease in incidence of CL after the introduction of ITNs versus no intervention and versus untreated nets.^19^ Although the majority of these studies demonstrated the potential for ITNs, IRS, and EM to impact on CL incidence, many had methodological limitations.^9^ Further, as diversity in the ecology of CL is large, even internally valid study results may not be generalizable to other ecological zones. There is little data demonstrating the relative effect sizes of these interventions in study settings, which might be directly relevant to the Moroccan environment. Though IRS was previously used in malaria control in Morocco, it has never been national policy to use IRS or ITNs for CL control in the country. This paper describes the conduct and results of a study to compare the relative efficacy and cost-effectiveness of IRS and long-lasting insecticidal nets (LLINs) relative to standard of care environmental management (SoC-EM) in a large cluster-randomized trial in Morocco.

## Methods

**Figure 1.**
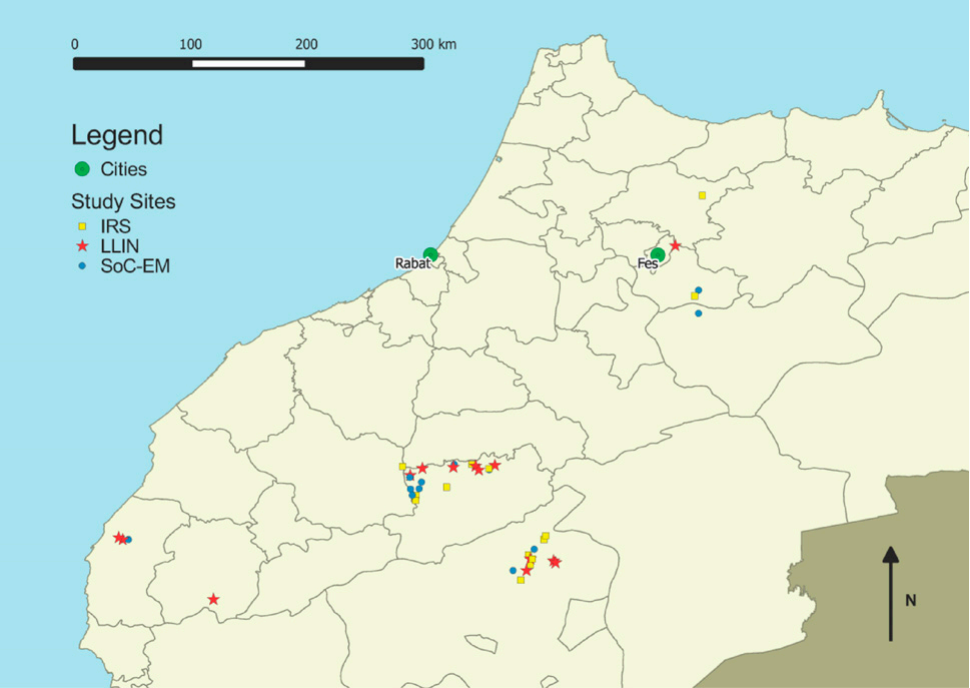
Map of study locations.

A three-arm cluster-randomized control trial comparing EM alone with EM combined with IRS and with EM combined with ITNs, stratified by baseline incidence of CL, was conducted over a period of 5 years (2 pre-intervention and 3 post-intervention) in 42 villages in Morocco (see Figure 1). Random allocation was conducted in 2009 prior to the rollout of interventions.

### Study site.

Villages were selected according to the following criteria. The inclusion criteria were at least three confirmed cases of CL in 2008, an incidence of at least 5 CL cases per 1,000 persons per annum in 2008, and a village population of at least 100. Villages were excluded if they were located in urban areas, rural localities with populations > 2,500, or had previous ITN distribution or IRS activities.

The selected villages were located in eight different districts covering different ecological zones: Boulmane, Sefrou, Taounate, My Yacoub, Essaouira, Chichaoua, Azilal, and Tinghir (Figure 1). The total population of these localities was 27,277, with a mean population size of 634 and an average CL incidence of 5.9 per 1,000 in 2008–2009. A comprehensive census was carried out in every village to identify the number of houses and sleeping units to be covered. All but six villages were at least 5 km from any other village. Of those within 5 km of one another, only two received allocations to a different study arm to that of their closest neighbor.

### Interventions.

Villages (clusters) were randomly allocated to one of the following interventions:

1. Standard EM consisted of campaigns to promote personal protection against exposure to sandflies, cleaning of animal sheds, waste disposal, and maintenance of hygiene (SoC-EM).
2. IRS using α-cypermethrin (10% SC [suspension concentrate] with a target dose of 0.03 g/m^2^ was conducted. All indoor surfaces, including roof structures, animal shelters, and caves near houses, were sprayed during one spray round in June of each intervention year plus SoC-EM. Spray coverage was determined for each village from spray program reports compiled by spray supervisors for each intervention year.
3. Distribution of ITNs or LLINs to cover all sleeping areas, before the start of transmission in year 1, and maintained as needed each year thereafter, plus SoC-EM. ITNs were impregnated with deltamethrin at 55 mg/m^2^. LLINs were Permanet2^®^ (Vestergaard Frandsen, Lausanne, Switzerland).

In addition, information and education campaigns (IEC) were conducted to sensitize communities involved in the trial and to improve use and adherence to the interventions, e.g., continuous and correct use of ITN/LLINs, provision of access to spray teams and avoidance of replastering/repainting or washing of walls subsequent to spraying.

### Sample size.

Sample size estimation was based on the design principles of cluster-randomized trials.^20^ A total of 14 clusters per study arm were required under the following assumptions:

1. Mean baseline CL incidence (all age groups) in areas under EM alone of 7 per 1,000 per year based on incidence in the study area for the year mid-2008 to mid-2009.
2. Three year post-intervention follow-up.
3. Effect of IRS or LLIN plus EM versus EM alone to result in at least 50% lower incidence than in the EM-only arm. Surveillance data from Morocco in areas where ITNs have been introduced previously, suggested that a 50% reduction in incidence would be realistic. An effect of less than 50% reduction was regarded as too small to justify the intervention, given the resources required.
4. Mean number of persons of all ages followed up per cluster = 750.
5. Coefficient of variation between clusters = 0.5.
6. Power = 80%; significance = 5% (α).

### Outcomes.

#### CL incidence.

The primary outcome measure of the trial was CL incidence. Cases of CL were monitored routinely in all study villages, both by passive and by active surveillance. Each identified case was confirmed by direct microscopic examination of dermal scrapings from CL consistent lesions, and data recorded in leishmaniasis registers.

#### Passive surveillance.

Leishmaniasis registers are routinely used in Morocco. Individual records from these registers were entered into a central study data base. The village of origin for each case was entered in the data base so that cases could be appropriately allocated to their respective study arms. Copies of register pages were taken (by digital camera) at three monthly intervals, for entry into the database. Non-autochthonous cases were identified from the database and excluded from subsequent analysis.

#### Active surveillance.

House-to-house campaigns were conducted in March, September, and December in each study year to identify additional cases in the population. Active case registers were collected centrally and individual data entered into the study data base, after checking for duplication of cases with passive registers.

Taking into account seasonality and the transmission period in Morocco, data were summarized annually for the period July to June.

### *Phlebotomus* spp. abundance.

Sandfly abundance was measured in eleven localities; Tabia, Aderdour, Soualeh, and Ait Chribou in the LLIN arm; Ait Chaib, Ait Boukidor, and M'Rouj in the IRS arm; and Aichoun, Bousdouk, Azrou, and Bouassem in the SoC-EM arm. Systematic sandfly collections, using the sticky trap method (20 × 30 cm papers coated with castor oil), were carried out bimonthly inside animal shelters from April to November 2011 and 2012.^21^ Five animal shelters were chosen at random in each locality and 10 traps were placed in each station before sunset and collected the following day. All sand flies were sorted and assigned to species based on morphological characteristics using standard identification keys.^22^ The mean abundance of flies per night was calculated by study arm, for 2011 and 2012.

### Costing.

In addition to epidemiological and entomological data collection, detailed cost and cost-effectiveness analyses were conducted. Details of methods are presented in the supplemental information.

### Coverage of interventions.

#### Indoor residual spraying.

IRS coverage was monitored by spray program reports during house-to-house censuses conducted during each annual spray round. Over the three spray rounds included in the study, household coverage was estimated to be 94%. There was little variation year to year. Supplemental Table 1 in the supplemental information shows spray coverage by study cluster.

#### Insecticide-treated bed nets/long-lasting insecticidal nets.

Because of the delay in delivery of LLINs for the trial, during the first intervention year of the study, a combination of LLINs and ITNs was distributed during June 2010. In the LLIN study arm, 95% of inhabitants lived in houses which received bed nets. During 2011, all ITNs were replaced by LLINs in all the localities involved in the study. From May to June of 2012, a household survey was conducted on a random sample of 10% of houses in each LLIN village to determine the availability and usage rate of nets. Supplemental Table 2 in the supplemental information shows that although ownership of nets was high (94% of households had a net), only 34% of the study population reported using a net at the time of the survey.

#### Environmental management.

Standard environmental practices were conducted in all study clusters. All interventions were conducted at the community level, consequently household and individual coverage was not measured.

### Statistical analysis.

Data were analyzed using Stata version 13.1 (STATA Corp., College Station, TX). Intention to treat analysis was carried out comparing CL incidence between study arms as randomized. Individual level Poisson regression was carried out with CL as response and treatment arm as explanatory variable. A separate analysis was done on the 2012 incidence data of the LLIN localities to determine if there was an association between LLIN use and CL incidence.

Sandfly counts per night were analyzed using a Poisson model with sticky trap area as the exposure (offset) to investigate whether sandfly densities differed between study arms and whether the intervention effect on sandfly density differed significantly between collection months (April to November), for the years 2011 and 2012 during which sandfly abundance was measured in a subset of localities. All analyses took account of within-cluster correlation of responses by using robust variance estimators as implemented in the *svy:* command in Stata (STATA Corp.).

### Ethics.

Data on health outcomes were derived from routine surveillance activities (both passive and active), which was de-identified and aggregated prior to analysis for this study. All study activities were reviewed and approved by the Ministry of Health (MoH), Morocco. The study was also monitored by an inter-sectoral committee consisting of representatives from the national, district, and community level. All study participants provided informed consent for participation in the research.

## Results

### Incidence of CL.

During the study period from July 1, 2008 to June 30, 2013, a total of 670 confirmed cases of CL were reported in the study area, of which 39% were by active case detection. Of all cases, 376 were reported in the pre-intervention period (years 1 and 2), and 294 cases were reported in the post-intervention period. Overall incidence was 4.1 per 1,000 cases per annum; this declined from 5.7 per 1,000 per annum (range by cluster 0–24 per 1,000 per annum) in the pre-intervention period (July 2008 to June 2010) to 3.0 per 1,000 per annum (range by cluster 0–20 per 1,000 per annum) in the post-intervention period (July 2010 to June 2013) (Tables 1 and 2). One cluster, despite meeting the inclusion criteria for CL incidence during the first half of 2008, had zero cases during the pre-intervention period. Incidence by study arm and by year showed year to year variations, but with a general downward trend in incidence (Table 1). Incidence in the LLIN and IRS arm of the study is somewhat higher than in the control arm during the pre-intervention period, but lower than in the control arm after the start of IRS and the distribution of nets.

In Table 2, incidence is summarized by pre- and post-intervention period, and by study arm. This shows that mean incidence was comparable in the three study arms in the pre-intervention period. In the post-intervention period, there was a sharp decline in incidence in the IRS arm of the study, with some variation between the three intervention years, but with an overall reduction corresponding to an incidence rate ratio (IRR) relative to the control arm of 0.31, (95% confidence interval [CI] = 0.14–0.67, *P* = 0.004). CL incidence in the LLIN arm was also lower than in the control arm after the distribution of nets, but the evidence for an intervention effect was weak with a nonsignificant overall IRR relative to control of 0.64 (95% CI = 0.31–1.33, *P* = 0.224).

Comparison of CL incidence in year 5 (2012–2013) in villages of the LLIN arm of the study in relation to village LLIN usage levels collected in the household survey conducted in 2012 showed that incidence was inversely related to LLIN use in the village, but this trend was not significant (IRR 0.91 per 10% increase in LLIN usage, 95% CI = 0.75–1.11, *P* = 0.32).

### Sandfly abundance.

During 2 years of capture (2011–2012), 10,325 sand flies were collected in 11 sites. Eleven different sandfly species were identified: *Ph. sergenti*, *Ph. longicuspis*, *Ph. perniciousis*, *Ph. papatasi*, *Ph. ariasi*, *Ph. chabaudi*, *Ph. alexandri, Sergentomyia minuta*, *S. fallax*, *S. dreyfusi*, and *S. antennata*. Except in Boukidour and Azrou, where it constituted only 21.3% and 32.3%, respectively, of total captures, *Ph. sergenti* was the most prevalent species: 87.0%, 50.4%, 49.4%, 73.8%, 64.9%, 46.5%, 47.9%, 44.7%, and 53.9% of total sand flies collected in Aichoun, Bouassem, Bousdouk, Ait Chaib, L'Mrouj, Tabia, Ait Chribou, Soualeh, and Aderdour, respectively.

Comparison of sandfly abundance per trapping night between study arms showed that mean abundance in the IRS villages was substantially lower than in the SoC-EM sites (Table 3), with abundance ratios of IRS versus control of 0.47 (95% CI = 0.21–1.03, *P* = 0.059) and 0.37 (95% CI = 0.16–0.86, *P* = 0.025) in 2011 and 2012, respectively, and 0.39 (95% CI = 0.18–0.85, *P* = 0.022) for the IRS effect over the 2 years combined. The difference in sandfly abundance between the LLIN arm and the control villages was not significant.

### Cost and cost-effectiveness.

#### Costs.

The total costs of the interventions broken down by health system level and the numbers of persons protected in the experimental arms are presented in the supplemental information in Supplemental Table 3. The total for the two arms was similar with the LLIN arm being slightly less costly than the IRS arm. The IRS arm also protected fewer individuals. Thus the cost per person-year of protection for IRS was higher than the cost per person-year of protection for LLINs. These estimates reflect community level protection offered rather than individual protection based on living in a house which was sprayed or owned and used LLINs.

The costs of the interventions were largely related to the distribution of the commodities themselves (IRS: 95% delivery, 5% commodity; LLINs: 85% delivery, 15% commodity). The actual LLINs and insecticides for IRS represented relatively small amounts of the total cost (Supplemental Table 4).

The total economic costs of the LLIN arm were estimated to be approximately U.S. dollars (USD) 244,832. The total economic costs of the IRS arm were estimated to be approximately USD 260,405.

#### Cost-effectiveness.

The IRS intervention was estimated in base case scenario to have averted more cases of CL than the LLIN arm, and both averted cases relative to the SoC-EM arm (Table 4). No interventions were estimated to avert large numbers of disability adjusted life years (DALYs) in base case analysis, given the nonfatal nature of CL and the relatively low disability weight associated with the disease. IRS was estimated to be a relatively more cost-effective intervention than LLINs in base case analysis, while both interventions added incremental costs above the SoC-EM approach, they were also more effective than SoC-EM. Neither LLINs nor IRS met World Health Organization (WHO) criteria for being considered a cost-effective intervention in the Moroccan context (cost per DALY averted ≤ 3 × gross domestic product [GDP] per capita).

#### Sensitivity analysis.

The sensitivity analysis reinforced the conclusion that IRS was a more cost-effective intervention than LLINs for CL prevention in Morocco (Details in Supplemental Information; Supplemental Table 5 and Supplemental Figure 1), but also that both interventions would not be considered cost-effective by WHO standards (cost per DALY averted > 3 × GDP per capita). In areas with higher baseline incidence, and delivered as a routine intervention rather than in the context of a community-randomized trial, both IRS and LLINs may be cost-effective interventions in the Moroccan context.

## Discussion

Control measures against ACL in Morocco rely both on case management and vector control. Vector control methods have historically relied mainly on EM. In addition, focal IRS with DDT or synthetic pyrethroids and distribution of LLINs has been conducted by the National Leishmaniasis Control Program in some transmission foci. This study compared the effectiveness of these vector control methods in a cluster-randomized controlled trial.

The main findings indicate that both IRS with α-cypermethrin at 0.30 g/m^2^ and the use of LLINs reduced the incidence of CL, however, the reduction due to LLINs did not reach statistical significance and the protective effect size associated with IRS was much larger.

To our knowledge, only one study of IRS for CL prevention and no studies of LLIN for CL prevention have previously been conducted in Morocco.^23^ The one study of IRS found significant reductions in CL incidence but no change in sandfly abundance compared with control villages after 2 years of spraying pyrethroid IRS, though the study was conducted with only two intervention and control locations and therefore lacked statistical power.^24^ We are aware of only one study which compared ITN interventions to IRS interventions in areas with similar vectors to those found in Morocco. In an individual (household) block-randomized trial in an urban area (Kabul, Afghanistan), where the predominant vector is *Ph. sergenti*, ITNs and *chadors* showed the largest effects while IRS also appeared to be significantly protective.^12^

LLINs showed lower efficacy than IRS spraying in this trial. It is possible that this finding is due to differences in the susceptibility of sandfly vectors to the insecticides used in the trial. In the areas where this trial was conducted, *Ph. sergenti* is susceptible to α-cyhalothrin, whereas susceptibility to deltamethrin (the insecticide used on the LLINs) or α-cypermethrin (the insecticide used for IRS) was not tested, we expect that sandflies in the trial areas are also susceptible to both of these pyrethroid insecticides.^24^ Reported use of LLINs in the survey conducted in the final year of the study was low, possibly because the perceived risk of CL is small. Usage rates at the cluster level were inversely related to CL incidence (though the effect was not statistically significant), therefore, it is possible that the low levels of LLIN use compromised the efficacy of the LLIN intervention.

Although the use of LLINs was associated with lower vector abundance, these differences were smaller than those associated with IRS and were not statistically significant. The entomological findings were thus consistent with the epidemiological outcomes of the trial and lend credence to the overall conclusion of protective effect due to IRS, though the lack of pre-intervention abundance measures limits the internal validity of these measures.

Several studies have evaluated ITNs against *Leishmania* vectors in the Old Word, namely in Iran, Syria, Turkey, and Afghanistan, as well as in South America.^12^–^14^,^16^,^17^,^19^,^25^,^26^ Contrary to our results, all these studies have shown that pyrethroid-treated nets provide significant protection against sandfly bites and reduced the transmission of ACL. Despite the evident reduction of ACL incidence attributable to ITN use, no significant reductions in density of the local vectors were detected in the above studies suggesting that monitoring vector density is an insufficient parameter to predict any efficacy of ITNs on ACL transmission.

The costs of the two main interventions (LLINs and IRS) in the study were high compared with the deployment of these interventions in other settings and in routine use.^27^,^28^ This finding was not surprising given that the price level in Morocco is higher than in most of sub-Saharan Africa, where the majority of previous cost and cost-effectiveness studies of LLINs and IRS have been conducted. In addition, the majority of costs in this study were found to be related to the deployment and monitoring of the interventions, which is in contrast to most routine programs where the commodities associated with LLIN delivery and IRS constitute the largest share of program costs. As these interventions were delivered in the context of a community-randomized trial covering a small population, this is also an expected finding. Sensitivity analysis indicates that if the interventions were delivered as routine interventions to larger populations, the cost per unit would decrease significantly. To our knowledge, no other studies have attempted to quantify the cost-effectiveness of these two vector control interventions for the prevention of CL.

In the base scenario, neither LLINs nor IRS appeared to be cost-effective per DALY averted due to CL using WHO thresholds for CE, though IRS, despite higher costs, was shown to be significantly more cost-effective than LLINs. The incidence of CL in the study areas was low in general, and as such only a small number of total cases of CL were averted, leading to higher cost-effectiveness ratios. The use of microscopic diagnostic confirmation, which is known to have low sensitivity may bias our incidence estimates downward,^29^ but there is no reason to assume that this differed between study arms. Sensitivity analysis indicates that in areas of significantly higher CL incidence, the interventions could become cost-effective by WHO standards. In addition, there is considerable debate about the appropriate disability weighting and duration that should be applied to CL.^30^,^31^ Although CL is generally not a fatal disease, there are potential severe long-term outcomes that may arise even after an acute case has spontaneously resolved. Furthermore, after resolution of acute cases, permanent scarring may remain, which can be severely stigmatizing and adversely affect individuals' social standing, marriageability, and long-term earning potential. All of these factors could indicate that the appropriate disability weighting and duration are underestimated in our analysis leading to overly pessimistic cost-effectiveness calculations.

## Conclusions

IRS with α-cypermethrin and LLIN distribution both reduced the incidence of CL in Morocco. IRS was highly effective, whereas the evidence for LLIN effect was weak and not statistically significant. LLIN efficacy may have been reduced by low usage rates resulting from the low disease burden. Because of the high costs of the interventions in the study areas and the relatively small disease burden, IRS is recommended to be targeted to areas of relatively high CL incidence in Morocco.

## Supplementary Material

Supplemental Datas.

## Figures and Tables

**Table 1:** Incidence of leishmaniasis by active and passive case detection, by year and study arm, Morocco July 1, 2008 to June 30, 2013

Year	Study arm	Cases	Person-years	Incidence rate per 1,000	95% CI		IRR	95% CI		*P* value
1	Control	63	9,761	6.5	4.55	9.15	1	−	−	−
	LLIN	103	12,783	8.1	5.26	12.35	1.25	0.75	2.06	0.378
	IRS	73	10,198	7.2	4.35	11.77	1.11	0.64	1.93	0.708
2	Control	42	9,761	4.3	2.46	7.51	1	−	−	−
	LLIN	48	12,783	3.8	2.17	6.50	0.87	0.43	1.78	0.702
	IRS	47	10,198	4.6	2.54	8.36	1.07	0.51	2.25	0.853
3	Control	64	9,761	6.6	3.31	12.98	1	−	−	−
	LLIN	47	12,783	3.7	1.50	9.04	0.56	0.20	1.57	0.263
	IRS	27	10,198	2.6	1.27	5.54	0.40	0.16	1.01	0.052
4	Control	23	9,761	2.4	1.43	3.88	1	−	−	−
	LLIN	37	12,783	2.9	1.57	5.33	1.23	0.60	2.52	0.566
	IRS	14	10,198	1.4	0.53	3.56	0.58	0.22	1.55	0.272
5	Control	49	9,761	5.0	3.08	8.19	1	−	−	−
	LLIN	30	12,783	2.3	1.14	4.85	0.47	0.21	1.04	0.061
	IRS	3	10,198	0.3	0.06	1.50	0.06	0.01	0.28	0.001

CI = confidence interval; IRS = indoor residual spraying; IRR = incidence rate ratio; LLIN = long-lasting insecticidal net.

**Table 2:** Incidence rates of leishmaniasis by active and passive case detection, by study arm, pre- and post-intervention, Morocco July 1, 2008 to June 30, 2013

Before/after interventions	Study arm	Cases	Incidence rate per 1,000	95% CI		IRR	95% CI		*P* value
Before (years 1 and 2)	Control	105	5.38	4.03	7.17	1	−	−	−
	LLIN	151	5.91	4.22	8.27	1.10	0.73	1.64	0.64
	IRS	120	5.88	3.60	9.61	1.09	0.65	1.84	0.73
After (years 3, 4, and 5)	Control	136	4.64	2.75	7.84	1	−	−	−
	LLIN	114	2.97	1.62	5.45	0.64	0.31	1.33	0.22
	IRS	44	1.44	0.74	2.79	0.31	0.14	0.67	0.004

CI = confidence interval; IRS = indoor residual spraying; IRR = incidence rate ratio; LLIN = long-lasting insecticidal net.

**Table 3:** Sandfly abundance by year (2011 and 2012) and by intervention arm

Year	Study arm	Sandfly count, mean per night	Sandfly abundance, mean/ m^2^	Sandfly abundance ratio	95% CI		*P* value
2011	Control	55.4	11.6	1	−	−	−
	LLIN	64.8	13.3	1.17	0.35	3.9	0.777
	IRS	25.8	5.2	0.47	0.21	1.03	0.059
2012	Control	59.0	13.0	1	−	−	−
	LLIN	43.7	9.1	0.74	0.39	1.40	0.319
	IRS	22.0	4.4	0.37	0.16	0.86	0.025
2011/2012 combined	Control	57.2	12.3	1	−	−	−
	LLIN	54.4	11.2	0.91	0.38	2.19	0.823
	IRS	23.9	4.8	0.39	0.18	0.85	0.022

CI = confidence interval; IRS = indoor residual spraying; LLIN = long-lasting insecticidal net.

**Table 4:** Cost-effectiveness estimates for LLINs and IRS

	IRS	LLIN
Total cost	USD 260,405	USD 244,832
Total PYP	30,594	38,349
Total cases averted	125	82
Total DALYs averted	2.9	1.9
Total cost per PYP	USD 8.51	USD 6.38
Cost per case averted	USD 2,091	USD 2,981
Cost per DALY averted	USD 90,904	USD 129,589

DALY = disability adjusted life year; IRS = indoor residual spraying; LLINs = long-lasting insecticidal nets; PYP = person-year protection; USD = U.S. dollars.
