# Supplementary material for: Effectiveness and Cost of Insecticide-Treated Bed Nets and Indoor Residual Spraying for the Control of Cutaneous Leishmaniasis: A Cluster-Randomized Control Trial in Morocco
Source: Am J Trop Med Hyg. 2016 Mar 2;94(3):679–85. doi: 10.4269/ajtmh.14-0510 (PMC4775907; doi:10.4269/ajtmh.14-0510)
Supplement: Supplementary file 1 [file SD13.pdf]

SUPPLEMENTAL INFORMATION

Costing methods. In addition to epidemiological and entomological data collection, a detailed cost and cost-effectiveness analysis was conducted. Details of these methods are presented below.

Cost data collection tools and indicators. An ingredients approach was applied to the development of cost-collection tools. Key-informant interviews and record reviews were conducted to identify all of the activities and resources needed, which were expected to be, or were used during the course of the trial. Care was taken to exclude activities that were specifically related to research and not necessary for the provision or performance of the intervention; these included enhanced case finding and enhanced vector surveillance beyond what was necessary for routine use of environmental management (EM), indoor residual spraying (IRS), or long-lasting insecticidal nets (LLINs). A standardized instrument for the collection of resource quantities and prices was developed for use at the national (central), province, and locality levels. The instrument was used by staff of the Ministry of Health (MoH) division of vector control to collect information on resource usa at each level of the health system (in each of 8 provinces and 28 localities (all with LLIN or IRS interventions)). Financing information for contributions from international donors was collected from budget and expenditure records.

Other data sources. Where information on cost or resource use was unavailable, the missing information was supplemented with information from the WHO-CHOICE database.[1] Price information was supplemented with data from the National Ministry of Health, the Ministry of Planning, and budgets and records from the Division of Vector Control, Ministry of Health, Morocco.

Analysis of cost data. Resource use was quantified and valued in Moroccan dirhams (DHS) in the year during which the resource use occurred. Costs were converted to USD using the prevailing average exchange rate for the period.[2] All costs were valued in 2010 USD, after adjusting for inflation using the consumer price index (CPI) for Morocco.[3] Prices derived from the WHO-CHOICE database were converted from International dollars using a purchasing power parity to local currency ratio of (1 International dollar to DHS 4.99) for 2009.[1,4]

In all cases, economic costs are presented, these are also known as opportunity costs. As such, capital costs, including vehicles, buildings, LLINs, and spray equipment, were annualized and discounted using assumed lifetimes and a social discount rate of 3%.[5]

Cost and cost-effectiveness outcomes. Two main outcomes were measured, numbers of persons living in houses with vector control per year (or person-years of protection), an output measure, and incident cases of cutaneous leishmaniasis (CL) prevented. In addition, a cost per disability adjusted life year (DALY) averted was also calculated using disability weights and assumed disease duration consistent with the Global Burden of Disease estimates.[6]

Sensitivity analysis. A one way sensitivity analysis was conducted to determine the robustness of the cost and cost-effectiveness model to various assumptions made during the assessment. Parameters which were varied included discount rate, prices of LLINs and insecticides used, cost of worker days, costs of vehicle rental, allocation of shared costs, numbers of persons protected by the intervention, disability weight and duration, baseline incidence and the estimated protective efficacy of the interventions.

Cost and cost-effectiveness results. *Costs.* The total costs of the interventions broken down by health system level and the numbers of persons protected in the experimental arms are presented in Supplemental Table 3. The total for the two arms was similar with the LLIN arm being slightly less costly than the IRS arm. The IRS arm also protected fewer individuals. Thus the cost per person-year of protection for IRS was higher than the cost per person-year of protection for LLINs. These estimates reflect community-level protection offered rather than individual protection based on living in a house that was sprayed or owned and used LLINs.

The costs of the interventions were largely related to the distribution of the commodities themselves (IRS: 95% delivery, 5% commodity; LLINs: 85% delivery, 15% commodity). The actual LLINs and insecticides for IRS represented relatively small amounts of the total cost (Supplemental Table 4).

The total economic costs of the LLIN arm were estimated to be approximately USD 244,832. The total economic costs of the IRS arm were estimated to be approximately USD 260,405.

Cost-effectiveness. The IRS intervention was estimated in base case scenario to have averted more cases of CL than the LLIN arm, and both averted cases relative to the SoC-EM-alone arm (Table 4 in the main paper). No interventions were estimated to avert large numbers of DALYs in base case analysis, given the nonfatal nature of CL and the relatively low disability weight associated with the disease. IRS was estimated to be a relatively more cost-effective intervention than LLINs in base case analysis, while both interventions added incremental cost relative to SoC-EM and were more effective than SoC-EM alone. Neither LLINs nor IRS met World Health Organization (WHO) criteria for being considered a cost-effective intervention in the Moroccan context (cost per DALY averted < = 3 × GDP per capita).

Sensitivity analysis. The sensitivity analysis reinforced the conclusion that IRS was a more cost-effective intervention than LLINs for CL prevention in Morocco (Supplemental Table 5 and Supplemental Figure 1), but also that both interventions would not be considered cost-effective by WHO standards (cost per DALY averted < = 3 × GDP per capita per DALY). In areas with higher baseline incidence, and delivered as a routine intervention rather than in the context of a community-randomized trial, both IRS and LLINs may be cost-effective interventions in the Moroccan context.

REFERENCES FOR SUPPLEMENTAL INFORMATION

1. WHO-CHOICE DATABASE. Available at: http://www.who.int/choice/en/. Accessed December 15, 2014.
2. OANDA Historical Currency Conversion database. Available at: http://www.oanda.com/currency/historical-rates/. Accessed December 15, 2014.
3. Bank Al-Maghrib. Available at: http://www.bkam.ma/. Accessed December 15, 2014.
4. The World Bank Open Data. Available at: http://data.worldbank.org/. Accessed December 15, 2014.
5. Tan-Torres Edejer T, Baltussen R, Adam T, Hutubessy R, Acharya A, Evans DB, Murray CJL, eds. 2003. *Making Choices in Health: WHO Guide to Cost Effectiveness Analysis*. Geneva, Switzerland: World Health Organization.
6. World Health Organization, 2004. *Global Burden of Disease Disability Weights*. Available at: http://www.who.int/healthinfo/global_burden_disease/estimates/en/index2.html. Accessed December 15, 2014.
7. White MT, Conteh L, Cibulskis R, Ghani A, 2011. Costs and cost-effectiveness of malaria control interventions–a systematic review. *Malar J 10:* 337.

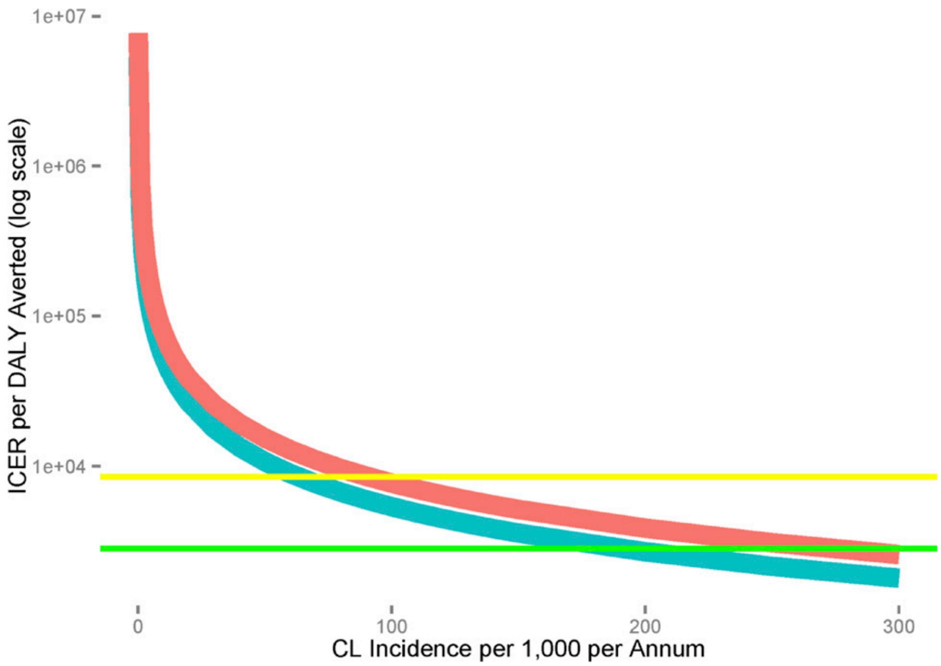

Supplemental Figure 1. Threshold analysis of CL incidence versus ICER.

Supplemental Table 1
IRS coverage by year and study cluster

Province	Locality	Household coverage (%) 2010	2011	2012
Azilal	Ait Ali Ben Salem	90.9	100.0	100.0
	Ait Sry	87.4	96.0	90.1
	Iammoumen	85.4	85.4	85.4
	Nzala	98.2	91.0	98.1
	Skoura	97.9	97.9	97.9
	Tanaghmalte	97.9	97.0	80.0
Tinghir	Ait Abdoune	98.1	96.0	96.0
	Ait Boukidour	97.8	99.0	99.0
	Ait Hammou Osaid	95.2	98.0	98.0
	Ait Ibrine	97.9	98.0	98.0
	Tarmouchte	100.0	98.0	98.0
	Tizguine	97.6	97.0	97.0
Taounate	M'Rouj	96.4	95.0	87.0
Sefrou	Ait Chaib	89.1	81.5	94.1
Total		94.3	94.6	92.9

IRS = indoor residual spraying.

Supplemental Table 2
ITN/LLIN ownership and usage by locality (2012)

Localities	NHH	NHH surveyed	N pop. surveyed	LLIN per HH	Percent HH with LLIN	Percent individuals who slept under LLIN the night before survey
Ait Chribou	131	13	85	1.85	95.5	25.9
Ait Waryiane	92	10	60	4.60	71.0	98.3
Bouaziare	120	12	80	3.75	87.4	32.5
Iaatarne	150	11	68	3.82	62.3	33.8
Waourinte	75	8	35	2.25	86.9	37.1
Aderdor	136	26	159	2.92	100.0	29.6
Soualeh	48	9	53	2.67	100.0	37.7
Tabia	375	42	209	2.88	100.0	28.7
Ouled Ayed	115	11	65	2.91	100.0	36.9
Ait Ali	176	41	354	2.83	100.0	20.6
Ait Boulemane	93	25	218	3.00	100.0	17.3
Ait Brahim	199	35	257	2.57	100.0	28.4
Jida	101	21	140	3.00	100.0	33.6
Timolite PAM	33	33	169	2.54	99.4	84.0
Total	1,833	299	1,964	2.89	94.4	34.3

ITN/LLIN = insecticide-treated bed nets/long-lasting insecticidal nets; NHH = number of households; N. pop = total population; HH = household.

Supplemental Table 3
Total economic costs of the IRS and LLIN interventions

	IRS	LLINs
Central	USD 87,852	USD 94,127
Provincial	USD 82,401	USD 81,635
Locality	USD 90,151	USD 69,071
Total	USD 260,405	USD 244,832

IRS = indoor residual spraying; LLIN = long-lasting insecticidal nets; USD = U.S. dollars.

Supplemental Table 4
Cost category distribution by health system level

		IRS (%)	LLIN (%)
Central	Recurrent	95	95
	Capital	5	5
Province	Recurrent	92	89
	Capital	8	11
Locality	Recurrent	92	38
	Capital	8	62
Percent commodity		5	15
Total	Recurrent	93	77
	Capital	7	23

IRS = indoor residual spraying; LLIN = long-lasting insecticidal nets.

SUPPLEMENTAL TABLE 5

One-way sensitivity analysis

Assumption	Base value	Change		Result		Rationale
		IRS	LLIN	IRS	LLIN	
Base scenario	All base values	NC		USD 8.51 pPYP	USD 6.38 pPYP	Not applicable
Discount rate	3%	Decrease to 0% or<br>Increase to 10%		Decrease<br>NC<br>Increase<br>USD 8.52	Decrease<br>USD 6.33<br>Increase<br>USD 6.52	Minimum and maximum plausible values
Price of LLINs	USD 4 or 5 each	NC	Increase to USD 15	NE	USD 8.46 pPYP	Estimate point at which LLIN cost pPYP is higher than IRS
Price of α-cypermethrin (10% SC)	DHS 300/L	Reduce to DHS 25	NC	USD 7.92 pPYP	NE	Estimate point at which IRS cost pPYP is less than LLIN
Cost of worker/ spray-man day	DHS 100	Increase to DHS 200/day<br>or Decrease to DHS 50/day		Increase<br>USD 9.13 pPYP<br>Decrease<br>USD 8.20 pPYP	Increase<br>USD 6.66 pPYP<br>Decrease<br>USD 6.24 pPYP	Minimum and maximum plausible values
Cost of vehicle rental	DHS 500/day or DHS 300/day	Decrease by 50% or<br>Increase by 100%		Decrease<br>USD 8.29 pPYP<br>Increase<br>USD 8.95 pPYP	Decrease<br>USD 6.26 pPYP<br>Increase<br>USD 6.93 pPYP	Range of estimates obtained locally
Allocation of central level shared costs	IRS (33%)<br>LLINs (35%)<br>SoC-EM (31%)	Increase share to 100%<br>Decrease share to 0%		Increase<br>USD 867 pPYP<br>Decrease<br>USD 5.64 pPYP	Increase<br>USD 671 pPYP<br>Decrease<br>USD 3.93 pPYP	Test assumption of cost sharing across interventions
Total PYP	IRS: 30,594<br>LLINs: 38,349	Increase by 50%<br>Decrease by 50%		Increase<br>USD 5.67 pPYP<br>Decrease<br>USD 17.02 pPYP	Increase<br>USD 4.26 pPYP<br>Decrease<br>USD 12.77 pPYP	Test whether error in population counts could affect decision
Cost per PYP	IRS: USD 8.51<br>LLIN: USD 6.38	Decrease to USD 6.70 for IRS and USD 2.20 for LLIN		USD 70,523 per DALY Averted	USD 44,686 per DALY Averted	Median estimates from[7]

DHS = Moroccan dirhams; IRS = indoor residual spraying; LLIN = long-lasting insecticidal net; NC = no change; NE = no effect; pPYP = per person-year protection; SC = suspension concentrate; SoC-EM = standard of care environmental management.
